# Application of Standardized Precipitation Index to assess meteorological drought in Bangladesh

**DOI:** 10.4102/jamba.v8i1.280

**Published:** 2016-09-30

**Authors:** Md. Anarul H. Mondol, Subash C. Das, Md. Nurul Islam

**Affiliations:** 1 Department of Geography and Environment, Jahangirnagar University, Bangladesh

## Abstract

Bangladesh is one of the vulnerable countries of the world for natural disasters. Drought is one of the common and severe calamities in Bangladesh that causes immense suffering to people in various ways. The present research has been carried out to examine the frequency of meteorological droughts in Bangladesh using the long-term rainfall data of 30 meteorological observatories covering the period of 1948–2011. The study uses the highly effective Standardized Precipitation Index (SPI) for drought assessment in Bangladesh. By assessing the meteorological droughts and the history of meteorological droughts of Bangladesh, the spatial distributions of meteorological drought indices were also analysed. The spatial and temporal changes in meteorological drought and changes in different years based on different SPI month intervals were analysed. The results indicate that droughts were a normal and recurrent feature and it occurred more or less all over the country in virtually all climatic regions of the country. As meteorological drought depends on only rainfall received in an area, anomaly of rainfall is the main cause of drought. Bangladesh experienced drought in the years 1950, 1951, 1953, 1954, 1957, 1958, 1960, 1961, 1962, 1963, 1965, 1966, 1967 and 1971 before independence and after independence Bangladesh has experienced droughts in the years 1972, 1973, 1975, 1979, 1980, 1983, 1985, 1992, 1994, 1995, 2002, 2004, 2006, 2009 and 2011 during the period 1948–2011. The study indicated that Rajshahi and its surroundings, in the northern regions and Jessore and its surroundings areas, the island Bhola and surrounding regions, in the south-west region, were vulnerable. In the Sylhet division, except Srimongal, the areas were not vulnerable but the eastern southern sides of the districts Chittagong, Rangamati, Khagrachhari, Bandarban and Teknaf were vulnerable. In the central regions, the districts of Mymensingh and Faridpur were more vulnerable than other districts.

## Introduction

Drought generally characterises as a short-term meteorological occurrence, which stems from a shortage of rainfall for a long period of time comparing with its average and normal conditions (Mondol, Islam & Das 2013). It is a natural event that happens in all climatic zones all over the world (Wilhite 1997). In any single region, drought can hit with its many facets and thus it is considered as a complex natural phenomenon and the impacts are mostly multiple and severe (Eriyagama, Smakhtin & Gamage 2009). Long-term drought with extreme climatic events hampers agricultural sectors (Ministry of Environment and Forests 2005). It is said that on average, drought hits once in 2.5 years in the country (Adnan 1993; Erickson, Ahmad & Chowdhury 1993; Hossain 1990). It is historically proved that north-western regions of the country experience more drought causing greater damages to crops than the other regions of the country (Rahman 1995). The Chittagong Hill tracts are also drought prone and these regions experience dryness for at least 7 months of the year and mostly from November to May (Abedin, Habiba & Shaw 2012). The frequency and intensity have been continuously increasing in recent years and affect agricultural production (Zimmermann, Glombitza & Rothenberger 2010). Droughts are also a recurrent feature in Bangladesh and hamper plant growth, lead to loss of crop production and cause food shortages and starvation.

Though droughts are natural occurrences, their impact can be mitigated and their adverse effects may be minimised by using advanced knowledge and technology (Rothauge 1998; Das 2000). Timely information and forecasting regarding drought can minimise the extent, frequency and duration of drought.

By decreasing the drought-related losses of life and human suffering, the economic and environmental damage can also be minimised (Murad & Islam 2011). Drought had occurred 19 times in Bangladesh between 1960 and 1991 (Abedin *et al.* 2012). Rainfall variability over time and space make the unique characteristics of the climate of Bangladesh. The different climatic modellers forecast and argue that climate change and the cruel combination of rainfall variability cause more extreme floods and longer periods of droughts (Shahid & Khairulmaini 2009). Bangladesh is a victim to climate change–induced calamity (IPCC 2007). The different analyses show that for many years, the country has been going through strong anomalies in rainfall variability and often below-normal rainfall, associated with long dry spells through the season, which hamper the growing seasons (IRIN 2008). This research work extends on the research of Mondol, Das and Islam (2015) and in this respect tried to establish the drought profile of drought indices and the pattern of drought in different parts of Bangladesh from Standardized Precipitation Index (SPI). The results of this study will then be disaggregated and aggregated and analyses of the results will portray a clear picture of the drought pattern in Bangladesh.

### Research aim and objectives

The aim of the research is the application of SPI to assess the meteorological drought in Bangladesh. The main objectives of the research were:

- to assess the meteorological drought in Bangladesh
- to understand the history of meteorological drought in Bangladesh.

### Study area

Bangladesh is located in the tropics between 20° 34’ and 26° 38’ north latitudes and 88° 01 and 92° 41’ east longitudes. It is a low-lying and riverine country with a large mangrove forest and has a 710-km-long coastline in the northern coastal area of the Bay of Bengal (Banglapedia 2007). Natural disasters, such as floods, drought, tornadoes and tidal bores, affect the country almost regularly. The study area of this research covers 30 meteorological stations of Bangladesh Meteorological Department (BMD).

For better understanding of the meteorological drought, the total area of Bangladesh was divided into the following regions:

- drought in northern regions
- drought in southern and western regions
- drought in central regions
- drought in eastern regions.

**FIGURE 1:**
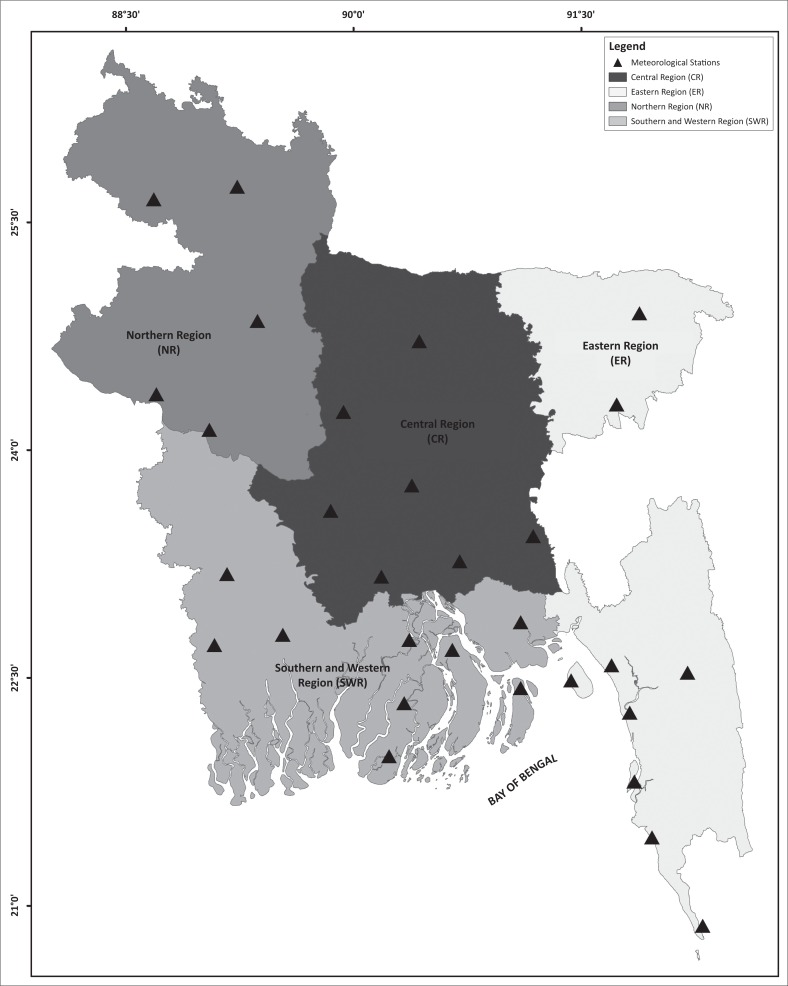
Study area and location of meteorological stations of Bangladesh for study (triangles).

The sub-divisions of the different parts of the analysis are shown in Figure 1.

## Data and methods

The data regarding rainfall in Bangladesh were obtained from the BMD. Monthly rainfall data over the country were collected for a maximum period of 64 years from 1948 to 2011.

### The calculation of Standardized Precipitation Index from rainfall data

The calculations of SPI explained by Giddings *et al.* (2005) were followed in this research. Firstly, the mean was calculated (Eqn 1):

$$Mean\;\bar{X}=\frac{\Sigma X}{N}$$ [Eqn 1]

Then the standard deviation of 1.0 was calculated (Eqn 2):

$$S=\sqrt{\frac{\Sigma (X-\bar{X})}{N}}$$ [Eqn 2]

The skewness was then calculated (Eqn 3):

$$\text{Skewness}=\frac{\text{N}}{\left(\text{N}-1)(\text{N}-2\right)}\sum {\left[\frac{X-\bar{X}}{S}\right]}^{2}$$ [Eqn 3]

After these, the rainfall data were transformed by the log (In) and then the mean was calculated. The Constant U, shape and scale were also calculated (Eqns 4, 5, 6 and 7):

$$logmean={\bar{X}}_{ln}=\frac{\ln(X)}{N}$$ [Eqn 4]

$$U=\ln(X)-{\bar{X}}_{ln}$$ [Eqn 5]

$$shape=\beta =\frac{1}{4U}\left[1+\sqrt{\frac{4U}{3}}\right]$$ [Eqn 6]

$$scale=\alpha =\frac{\bar{X}}{\beta }$$ [Eqn 7]

In this step, the gamma distribution was performed with the shape and scale values (Eqn 8):

$$Cumulative\;Gamma\;transform=G(x)=\frac{1}{\alpha^{\beta }\Gamma \beta }\int_{0}^{x}x^{\beta -1}e^{\frac{-x}{\alpha }}dx$$ [Eqn 8]

The gamma-transformed values were again transformed, with different formulas according to Eqns 9, 10, 11 and 12:

$$t\;transform=t=\sqrt{ln\left[\frac{1}{X_{g}}\right]}$$ [Eqn 9]

where *X_g_* ≤ 0.5

$$\text{or},\;t\;transform=t=\sqrt{ln\left[\frac{1}{1-X_{g}}\right]}$$ [Eqn 10]

where *X_g_* ≤ 1.0

$$SPI=-\left[t-\frac{c_{0}+c_{1}t+c_{2}t^{2}}{1+d_{1}t+d_{2}t^{2}+d_{3}t^{3}}\right]$$ [Eqn 11]

where *X_g_* ≤ 0.5

$$\text{or},\;SPI=+\left[t-\frac{c_{0}+c_{1}t+c_{2}t^{2}}{1+d_{1}t+d_{2}t^{2}+d_{3}t^{3}}\right]$$ [Eqn 12]

where *X_g_* ≤ 1.0.

Finally, SPI values were calculated (Eqn 12) with different formulas according to the magnitude of the gamma-transformed values:

Co = 2.5155517, C1 = 0.802853, C2 = 0.010328, D1 = 1.432788, D2 = 0.189269 and D3 = 0.001308.

The analysis of SPI was done using the Drought Index Calculator (DrinC) (Tigkas, Vangelis & Tsakiris 2014), which was developed by the Laboratory of Reclamation Works & Water Resources Management, National Technical University of Athens, and this software was downloaded from http://www.ewra.net/drinc on 01 June 2013. McKee *et al.* (1993) first defined the index and the criteria for drought classifications by SPI values. In this research, the modified classification of Mondol *et al.* (2015) (Table 1) was followed. The 3-month SPI (M_3) for the months of January – March, the 6-month SPI (M_6) for the months of January – June, the 9-month SPI (M_9) for the months of January – September and the 12-month SPI (M_12) for annual drought were analysed for the study.

**TABLE 1:** Drought category.

Standardized Precipitation Index values	Drought category
> 0.00	No drought
< 0.00–0.99	Normal drought
−1.00–1.49	Moderate droughts
−1.50–1.99	Severe droughts
−2.00 and less	Extreme drought

## Results and discussion

In this section, the results of the analysis are presented with a discussion, which highlights the drought assessment and major drought history of Bangladesh. Bangladesh is a drought-prone country. The meteorological drought is more prominent here and the people of Bangladesh are more familiar with agricultural drought.

### Meteorological drought in northern regions of Bangladesh

It is said that the northern regions of Bangladesh are more prone to drought. For assessment of the drought of the northern region, the meteorological information of Rangpur, Dinajpur, Bogra, Rajshahi and Ishurdi were analysed.

**FIGURE 2:**
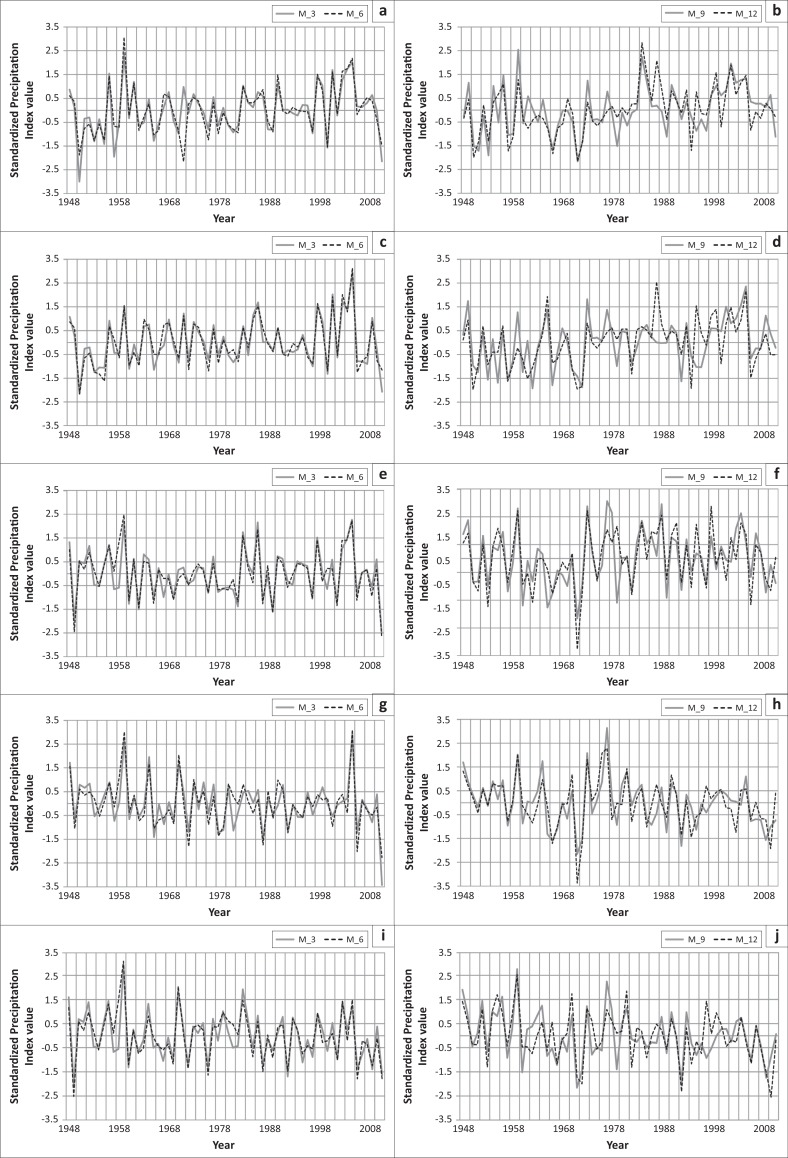
Drought in (a) Rangpur, (c) Dinajpur, (e) Bogra, (g) Ishurdhi and (i) Rajshahi based on 3-month SPI (M_3) and 6-month SPI (M_6). Drought in (b) Rangpur, (d) Dinajpur, (f) Bogra, (h) Ishurdhi and (j) Rajshahi based on 9-month SPI (M_9) and 12-month (annual) SPI (M_12).

For the short-term drought analysis based on 3-month SPI, from Figure 2, it is seen that 2 extreme droughts, 2 severe droughts, 3 moderate droughts and 24 normal droughts occurred in Rangpur region, and for medium and short-term drought analysis based on 6-month SPI, 1 extreme drought, 3 severe droughts, 4 moderate droughts and 27 normal droughts occurred in this area. In Dinajpur, 28 normal droughts occurred based on 3-month SPI; 1 extreme drought, 1 severe drought, 7 moderate droughts and 26 normal droughts occurred based on 6-month SPI. Seven severe droughts, 6 moderate droughts and 15 normal droughts based on 9-month SPI and 6 severe droughts, 4 moderate droughts and 19 normal droughts based on 12-month SPI had occurred (Figure 2). Bogra is also a drought-prone area of Bangladesh. In Bogra, 1 extreme drought, 3 severe droughts, 6 moderate droughts and 21 normal droughts based on 3-month SPI had occurred; 2 extreme droughts, 1 severe drought, 9 moderate droughts and 15 normal droughts based on 6-month SPI; 2 extreme droughts, 1 extreme drought, 3 severe droughts, 7 moderate droughts and 19 normal droughts based on 9-month SPI and 12-month SPI had occurred.

In Rajshahi 1 extreme drought, 3 severe droughts, 8 moderate droughts and 21 normal droughts had occurred based on the 3-month SPI; 1 extreme drought, 3 severe droughts, 7 moderate droughts and 18 normal droughts had occurred based on 6-month SPI; 1 extreme drought, 3 severe droughts, 4 moderate droughts and 27 normal droughts had occurred based on the 9-month SPI and 2 extreme droughts, 3 severe droughts, 5 moderate droughts and 21 normal droughts had occurred based on the 12-month SPI (Figure 2).

In Ishurdi, 1 extreme drought, 1 severe drought, 7 moderate droughts and 23 normal droughts had occurred based on the 3-month SPI; 2 extreme droughts, 2 severe droughts, 4 moderate droughts and 24 normal droughts had occurred based (Figure 2) on the 6-month SPI; 1 severe drought, 4 severe droughts, 5 moderate droughts and 22 normal droughts had occurred based on the 9-month SPI and 1 extreme drought, 3 severe droughts, 4 moderate droughts and 25 normal droughts had occurred based on the 12-month SPI.

From Figure 2, it is seen that mainly drought has occurred in the years 1949, 1953, 1954, 1957, 1958, 1960, 1962, 1963, 1965, 1966, 1967, 1968, 1969, 1972, 1974, 1976, 1978, 1981, 1982, 1985, 1987, 1989, 1992, 1995, 2000, 2002, 2008, 2009 and 2011. So it can be said that the northern parts of Bangladesh are prone to meteorological drought. In the years 1971, 1992, and 2010 extreme drought has occurred. In the years 1950, 1951, 1953, 1957, 1962, 1966, 1972, 1994, 2006 and 2009 severe meteorological droughts occurred in different parts of northern regions. During the years 1951, 1953, 1957, 1958, 1960, 1961, 1962, 1965, 1966, 1969, 1978, 1979, 1982 and 1997 moderate droughts occurred in this region (Figure 2). It is noted that droughts were recurrent and on average drought occurs once in every 2.5 years.

### Meteorological drought in the southern and western regions of Bangladesh

The southern and western regions of Bangladesh are also highly prone to drought. For the assessment of the drought of southern and western regions of Bangladesh, the meteorological information from Jessore, Satkhira, Khulna, Barisal, Patuakhali, Khepupara, Bhola, Hatyia, Maizdi Court and Sandwip were analysed.

**FIGURE 3:**
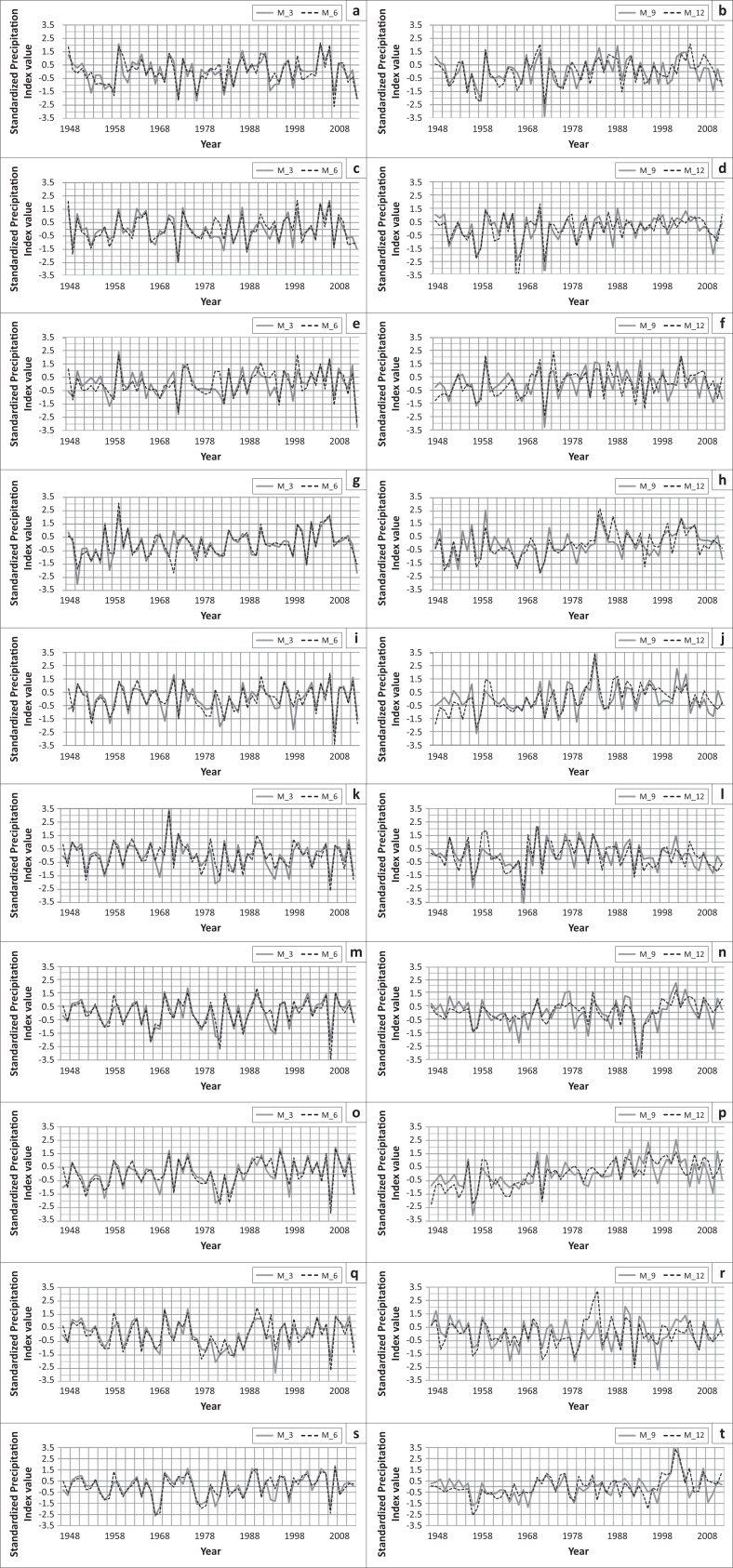
Drought in (a) Jessore, (c) Satkhira, (e) Khulna, (g) Barisal, (i) Patuakhali, (k) Bhola, (m) Hatyia, (o) Khepupara, (q) Maizdi Court and (s) Sandwip based on 3-month SPI (M_3) and 6-month SPI (M_6). Drought in (b) Jessore, (d) Satkhira, (f) Khulna, (h) Barisal, (j) Patuakhali, (l) Bhola, (n) Hatyia, (p) Khepupara, (r) Maizdi Court and (t) Sandwip based on 9-month SPI (M_9) and 12-month (annual) SPI (M_12).

The analysis shows that in Jessore 3 extreme droughts, 3 severe droughts, 6 moderate droughts and 14 normal droughts had occurred based on 3-month SPI; 3 extreme droughts, 2 severe droughts, 2 moderate droughts and 27 normal droughts had occurred based on 6-month SPI; 2 extreme droughts, 7 moderate droughts and 23 normal droughts had occurred based on 9-month SPI and 3 extreme droughts, 2 severe droughts, 5 moderate droughts and 19 normal droughts had occurred based on the 12-month SPI (Figure 3). The drought analysis for the meteorological station of Satkhira indicates that 1 extreme drought, 2 severe droughts, 8 moderate droughts and 26 normal droughts had occurred based on the 3-month SPI; 1 extreme drought, 2 severe droughts, 9 moderate droughts and 26 normal droughts had occurred based on 6-month SPI; 3 extreme droughts, 3 severe droughts, 3 moderate droughts and 17 normal droughts had occurred based on 9-month SPI and 3 extreme droughts, 4 moderate droughts and 21 normal droughts had occurred based on 12-month SPI. In Khulna, 2 extreme droughts, 2 severe droughts, 5 moderate droughts and 23 normal droughts had occurred based on 3-month SPI; 2 extreme droughts, 2 severe droughts, 5 moderate droughts and 26 normal droughts had occurred based on 6-month SPI; 1 extreme drought, 1 severe drought, 9 moderate droughts and 20 normal droughts had occurred based on 9-month SPI and 1 extreme drought, 3 severe droughts, 7 moderate droughts and 21 normal droughts had occurred based on 12-month SPI (Figure 3).

In Barisal, 2 extreme droughts, 2 severe droughts, 3 moderate droughts and 27 normal droughts had occurred based on 3-month SPI; 1 extreme drought, 3 severe droughts, 4 moderate droughts and 27 normal droughts had occurred based on 5-month SPI; 1 extreme drought, 5 severe droughts, 5 moderate droughts and 23 normal droughts had occurred based on 9-month SPI and 1 extreme drought, 4 severe droughts, 4 moderate droughts and 25 normal droughts had occurred based on 12-month SPI (Figure 3). The analysis of Bhola region drought indicates that in that place 1 extreme drought, 6 severe droughts, 4 moderate droughts and 17 normal droughts had occurred based on 3-month SPI; 1 extreme drought, 3 severe droughts, 5 moderate droughts and 21 normal droughts had occurred based on 6-month SPI; 2 extreme droughts, 4 moderate droughts and 27 normal droughts had occurred based on 9-month SPI and 1 extreme drought, 2 severe droughts, 7 moderate droughts and 23 normal droughts had occurred based on 12-month SPI (Figure 3).

In the island Hatiya, 2 extreme droughts, 3 severe droughts, 8 moderate droughts and 16 normal droughts had occurred based on 3-month SPI; 3 extreme droughts, 1 severe drought, 4 moderate droughts and 21 normal droughts had occurred based on 6-month SPI; 2 extreme droughts, 2 severe droughts, 8 moderate droughts and 17 normal droughts had occurred based on 9-month SPI and 1 extreme droughts, 2 moderate droughts and 26 normal droughts had occurred based on 12-month SPI (Figure 3). In Khepupara, 2 extreme droughts, 5 severe droughts, 4 moderate droughts and 18 normal droughts had occurred based on 3-month SPI; 3 extreme droughts, 2 severe droughts, 3 moderate droughts and 24 normal droughts had occurred based on 6-month SPI; 1 extreme drought, 1 severe drought, 4 moderate droughts and 32 normal droughts had occurred based on 9-month SPI and 3 extreme droughts, 4 severe droughts, 5 moderate droughts and 17 normal droughts had occurred based on 12-month SPI (Figure 3).

In Maizdi Court, 2 extreme droughts, 2 severe droughts, 8 moderate droughts and 20 normal droughts had occurred based on 3-month SPI; 1 extreme drought, 2 severe droughts, 11 moderate droughts and 20 normal droughts had occurred based on 6-month SPI; 4 extreme droughts, 1 severe drought, 6 moderate droughts and 19 normal droughts had occurred based on 9-month SPI and 1 extreme drought, 3 severe droughts, 7 moderate droughts and 21 normal droughts had occurred based on 12-month SPI. In Patuakhali, 3 extreme droughts, 2 severe droughts, 6 moderate droughts and 19 normal droughts had occurred based on 3-month SPI; 1 extreme drought, 4 severe droughts, 4 moderate droughts and 20 normal droughts had occurred based on 6-month SPI; 1 extreme drought, 3 severe droughts, 4 moderate droughts and 27 normal droughts had occurred based on 9-month SPI and 1 extreme drought, 4 severe droughts, 3 moderate droughts and 27 normal droughts had occurred based on 12-month SPI (Figure 3).

Like Hatyia, in the island Sandwip 1 extreme drought, 5 severe droughts, 5 moderate droughts and 15 normal droughts had occurred based on 3-month SPI; 3 extreme droughts, 2 severe droughts, 4 moderate droughts and 21 normal droughts had occurred based on 6-month SPI; 4 severe droughts, 5 moderate droughts and 22 normal droughts had occurred based on 9-month SPI and 1 extreme drought, 2 severe droughts, 5 moderate droughts and 12 normal droughts had occurred based on 12-month SPI (Figure 3).

The important drought years were 1957, 1958 and 1972 when extreme drought occurred; in 1955 and 1989 severe drought occurred; in the years 1951, 1962, 1966, 1975 and 1976 moderate drought occurred and in 1952, 1956, 1960, 1961, 1963, 1967, 1977, 1980, 1982, 1985, 1992, 1994, 1995, 1997, 1998, 1999, 2001, 2010 and 2011 slight drought occurred. So from the above discussion, it can be said that this area is also a drought-prone area of Bangladesh. In south-west regions, Jessore, Satkhira and its surroundings area, Bhola, Patuakhali and the surrounding regions were vulnerable. From the above discussion, it is seen that extreme drought occurred in the years 1957, 1971, 1972, 1966 and 1972 in some areas of these regions. In the years 1950, 1951, 1957, 1958, 1966, 1967, 1979, 1982, 1987, 1989 and 1994 severe drought occurred in some parts of these regions. Moderate droughts occurred in the years 1948, 1949, 1951, 1953, 1958, 1966, 1967, 1984, 1994, 1997, 2006, 2007 and 2011 in some parts of the regions (Figure 3). So it can be said that drought is a recurrent phenomenon in these regions.

### Meteorological drought in central regions of Bangladesh

The central regions of Bangladesh are not prominent in drought. For the assessment of drought of the central regions, the meteorological information of Dhaka, Tangail, Mymensingh, Faridpur, Madaripur, Chandpur and Comilla were analysed.

In Dhaka, 1 extreme drought, 4 severe droughts, 7 moderate droughts and 18 normal droughts occurred based on 3-month SPI; 4 extreme droughts, 1 severe drought, 6 moderate droughts and 17 normal droughts had occurred based on 6-month SPI; 2 extreme droughts, 3 severe droughts, 6 moderate droughts and 20 normal droughts had occurred based on 9-month SPI and 2 extreme droughts, 1 severe drought, 7 moderate droughts and 23 normal droughts had occurred based on 12-month SPI. In Tangail, 1 extreme drought, 5 severe droughts, 4 moderate droughts and 23 normal droughts had occurred based on 3-month SPI; 1 extreme drought, 3 severe droughts, 7 moderate droughts and 21 normal droughts had occurred based on 6-month SPI; 1 extreme drought, 5 severe droughts, 5 moderate droughts and 23 normal droughts had occurred based on 9-month SPI and 3 severe droughts, 9 moderate droughts and 29 normal droughts had occurred based on 12-month SPI.

**FIGURE 4:**
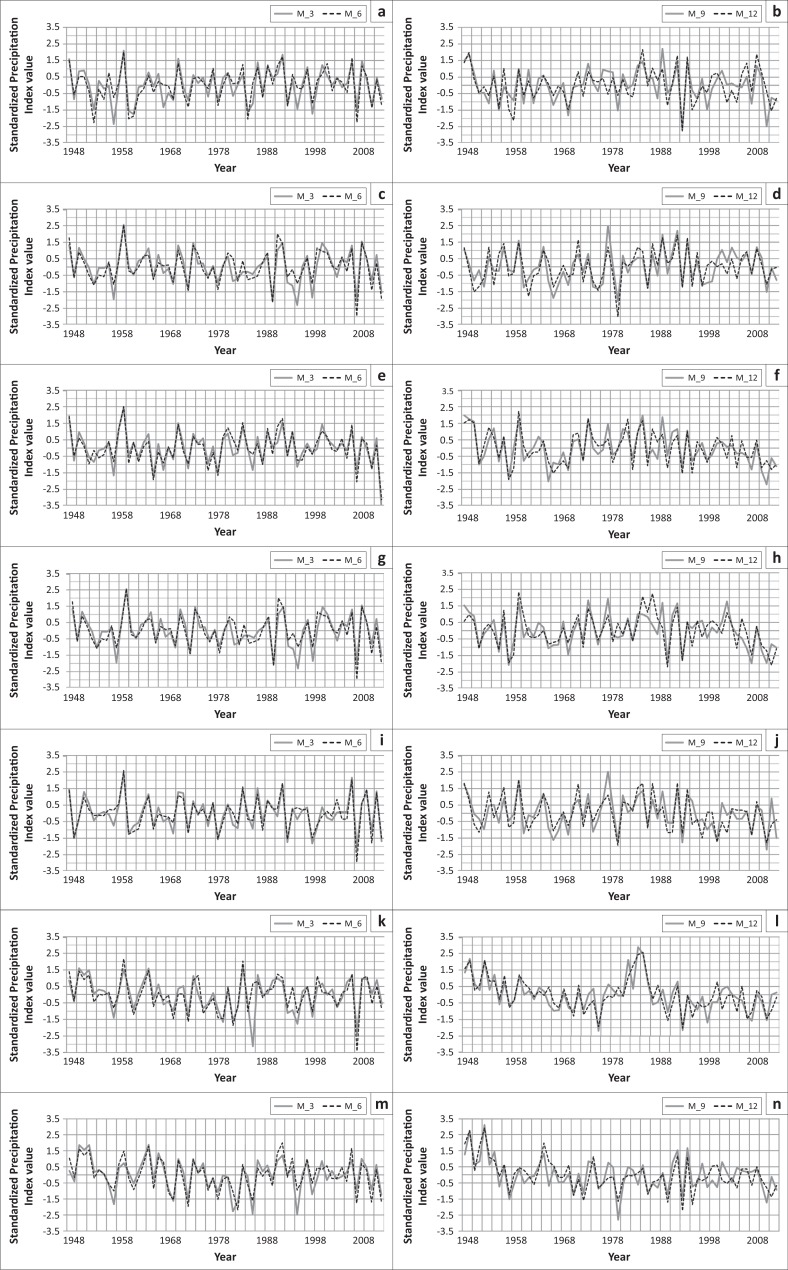
Drought in (a) Dhaka, (c) Mymensingh, (e) Faridpur, (g) Madaripur, (i) Tangail, (k) Chandpur and (m) Comilla based on 3-month SPI (M_3) and 6-month SPI (M_6). Drought in (b) Dhaka, (d) Mymensingh, (f) Faridpur, (h) Madaripur, (j) Tangail, (l) Chandpur and (n) Comilla based on 9-month SPI (M_9) and 12-month (annual) SPI (M_12).

The central northern part of the central region of Mymensingh is a drought-prone region where 1 extreme drought, 6 severe droughts, 3 moderate droughts and 20 normal droughts had occurred based on 3-month SPI; 3 extreme droughts, 1 severe drought, 6 moderate droughts and 19 normal droughts had occurred based on 6 month SPI; 1 extreme drought, 2 severe droughts, 10 moderate droughts and 21 normal droughts had occurred based on 9-month SPI and 1 extreme drought, 2 severe droughts, 9 moderate droughts and 19 normal droughts had occurred based on 12-month SPI (Figure 4). In Madaripur, 3 extreme droughts, 3 severe droughts, 4 moderate droughts and 18 normal droughts had occurred based on 3-month SPI; 2 extreme droughts, 2 severe droughts, 5 moderate droughts and 22 normal droughts had occurred based on 6-month SPI; 1 extreme drought, 4 severe droughts, 6 moderate droughts and 20 normal droughts had occurred based on 9-month SPI and 2 extreme droughts, 2 severe droughts, 8 moderate droughts and 17 normal droughts had occurred based on 12-month SPI (Figure 4).

In the central southern part of the central region of Faridpur 1 extreme drought, 3 severe droughts, 6 moderate droughts and 20 normal droughts had occurred based on 3-month SPI; 2 extreme droughts, 2 severe droughts, 2 moderate droughts and 26 normal droughts had occurred based on 6-month SPI; 2 extreme droughts, 1 severe drought, 6 moderate droughts and 27 normal droughts had occurred based on 9-month SPI and 4 severe droughts, 9 moderate droughts and 17 normal droughts had occurred based on 12-month SPI (Figure 4). In Chandpur, 2 extreme droughts, 3 severe droughts, 5 moderate droughts and 17 normal droughts had occurred based on 3-month SPI; 1 extreme drought, 2 severe droughts, 8 moderate droughts and 19 normal droughts had occurred based on 6-month SPI; 2 extreme droughts, 3 severe droughts, 2 moderate droughts and 25 normal droughts had occurred based on 9-month SPI and 1 extreme drought, 2 severe droughts, 7 moderate droughts and 27 normal droughts had occurred based on 12-month SPI (Figure 4).

In Comilla, 1 extreme drought and 38 normal droughts had occurred based on 1-month SPI; 3 extreme droughts, 3 severe droughts, 4 moderate droughts and 18 normal droughts had occurred based on 3-month SPI; 1 extreme drought, 7 severe droughts, 3 moderate droughts and 22 normal droughts had occurred based on 6-month SPI; 1 extreme drought, 3 severe droughts, 4 moderate droughts and 23 normal droughts had occurred based on 9-month SPI and 1 extreme drought, 4 severe droughts, 4 moderate droughts and 28 normal droughts had occurred based on 12-month SPI (Figure 4).

In the central regions, drought occurred mainly in the years 1950, 1957, 1958, 1961, 1992, 1979, 1989, 1994, 2006 and 2010. Moderate drought occurred in some parts of these areas during the years 1951, 1954, 1955, 1957, 1966, 1967, 1969, 1975, 1982, 1985, 1989, 1994, 2004, 2003, 2009 and 2010. Thus, it can be said that drought is also a recurrent feature in this regions (Figure 4). The meteorological drought is dynamic and changes in temporal and spatial extents also.

### Meteorological drought in the eastern regions of Bangladesh

Though drought is not more prominent in eastern parts of Bangladesh, sometimes meteorological drought occurs in these regions. The droughts in recent years were less compared to previous years. For assessment of the drought of eastern regions of Bangladesh, the meteorological information of Sylhet, Srimongal, Shitakundu, Chittagong, Rangamati, Kutubdia, Cox’s Bazar, Teknaf stations were analysed. The meteorological droughts of these areas are discussed below.

**FIGURE 5:**
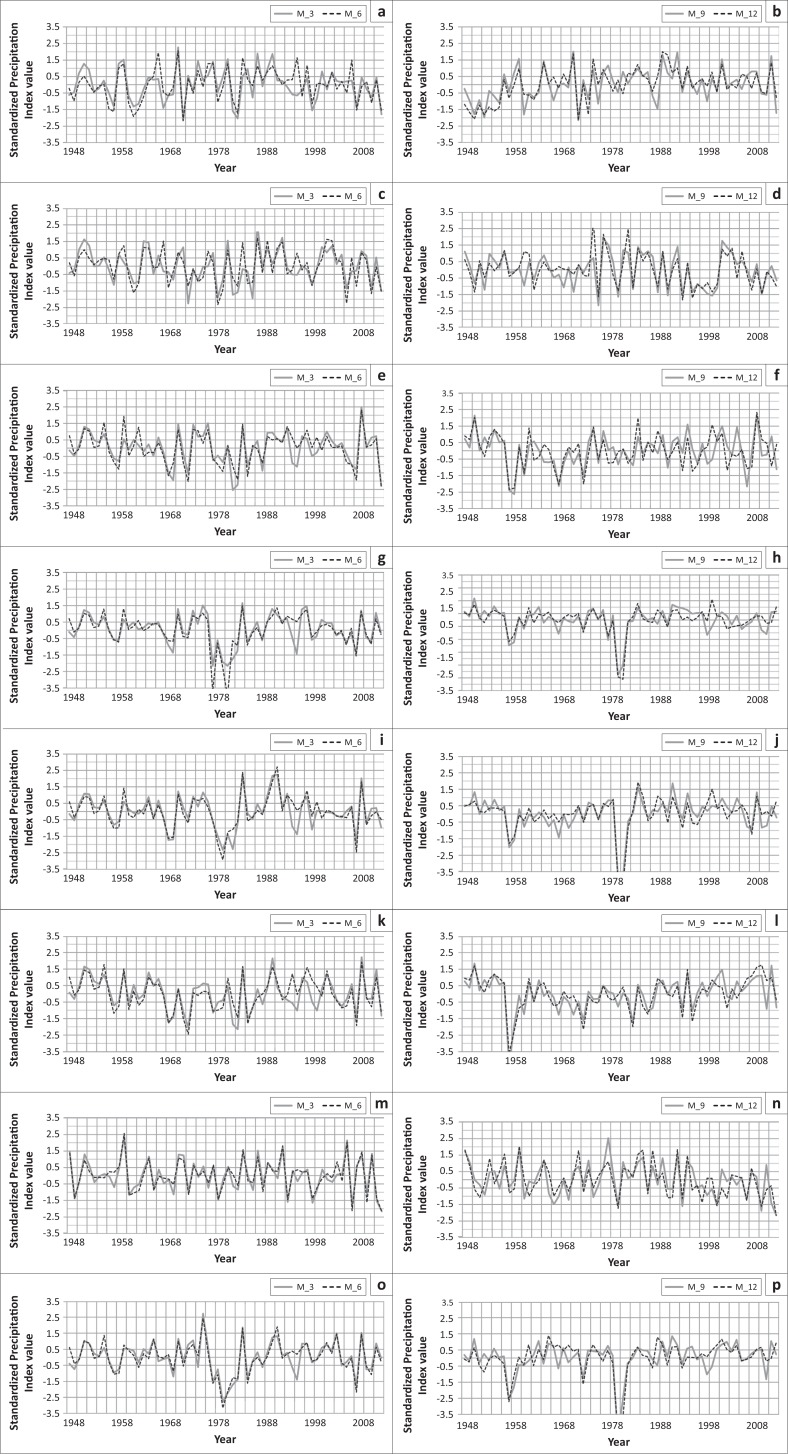
Drought in (a) Sylhet, (c) Srimangal, (e) Chittagong, (g) Khutubdia, (i) Sithakunda, (k) Rangamati, (m) Teknaf and (o) Cox”s Bazar based on 3-month SPI (M_3) and 6-month SPI (M_6). Drought in (b) Sylhet, (d) Srimangal, (f) Chittagong, (h) Khutubdia, (j) Sithakunda, (l) Rangamati, (n) Teknaf and (p) Cox”s Bazar based on 9-month SPI (M_9) and 12-month (annual) SPI (M_12).

In Sylhet, the heavy rainfall zone of Bangladesh, 2 extreme droughts, 3 severe droughts, 5 moderate droughts and 21 normal droughts had occurred based on 3-month SPI; 1 extreme drought, 5 severe droughts, 6 moderate droughts and 18 normal droughts had occurred based on 6-month SPI; 1 extreme drought, 4 severe droughts, 4 moderate droughts and 22 normal droughts had occurred based on 9-month SPI and 2 extreme droughts, 4 severe droughts, 4 moderate droughts and 20 normal droughts had occurred based on 12-month SPI (Figure 5). In Srimongal, the highest rainfall area, 1 extreme drought, 3 severe droughts, 7 moderate droughts and 24 normal droughts had occurred based on 3-month SPI; 2 extreme droughts, 3 severe droughts, 8 moderate droughts and 18 normal droughts had occurred based on 6-month SPI; 1 extreme drought, 4 severe droughts, 10 moderate droughts and 12 normal droughts had occurred based on 9-month SPI and 3 extreme droughts, 11 moderate droughts and 19 normal droughts had occurred based on 12-month SPI (Figure 5).

In the south-eastern part of Chittagong, 3 extreme droughts, 3 severe droughts, 5 moderate droughts and 17 normal droughts had occurred based on 3-month SPI; 2 extreme droughts, 4 severe droughts, 4 moderate droughts and 18 normal droughts had occurred based on 6-month SPI; 4 extreme droughts, 1 severe drought, 3 moderate droughts and 21 normal droughts had occurred based on 9-month SPI and 4 extreme droughts, 6 moderate droughts and 21 normal droughts had occurred based on 12-month SPI (Figure 5). In Kutubdia, 2 extreme droughts, 2 severe droughts, 4 moderate droughts and 22 normal droughts had occurred based on 3-month SPI; 3 extreme droughts, 1 severe drought and 24 normal droughts had occurred based on 6-month SPI; 2 extreme droughts, 3 severe droughts, 3 moderate droughts and 15 normal droughts had occurred based on 9-month SPI and 2 extreme droughts, 1 severe drought, 2 moderate droughts and 19 normal droughts had occurred based on 12-month SPI (Figure 5). The adjacent part of the Chittagong region, Sitakunda experienced 2 extreme droughts, 4 severe droughts, 3 moderate droughts and 22 normal droughts based on 3-month SPI; 2 extreme droughts, 3 severe droughts, 3 moderate droughts and 26 normal droughts had occurred based on 6-month SPI; 3 extreme droughts, 1 severe drought, 1 moderate drought and 21 normal droughts had occurred based on 9-month SPI and 2 extreme droughts, 1 severe drought, 2 moderate droughts and 20 normal droughts had occurred based on 12-month SPI (Figure 5).

The major part of Rangamati experienced 2 extreme droughts, 3 severe droughts, 7 moderate droughts and 20 normal droughts based on 3-month SPI; 1 extreme drought, 3 severe droughts, 6 moderate droughts and 22 normal droughts had occurred based on 6-month SPI; 1 extreme drought, 3 severe droughts, 5 moderate droughts and 20 normal droughts had occurred based on 9-month SPI and 3 extreme droughts, 2 severe droughts, 2 moderate droughts and 24 normal droughts had occurred based on 12-month SPI (Figure 5).

In Cox’s Bazar, 2 extreme droughts, 3 severe droughts, 4 moderate droughts and 23 normal droughts had occurred based on 3-month SPI; 3 extreme droughts, 2 severe droughts, 6 moderate droughts and 19 normal droughts had occurred based on 6-month SPI; 3 extreme droughts, 1 severe drought, 3 moderate droughts and 18 normal droughts had occurred based on 9-month SPI and 3 extreme droughts, 2 severe droughts and 21 normal droughts had occurred based on 12-month SPI (Figure 5). In the last south-eastern meteorological station and area Teknaf, 1 extreme drought, 4 severe droughts, 5 moderate droughts and 24 normal droughts had occurred based on 3-month SPI; 2 extreme droughts, 1 severe drought, 7 moderate droughts and 21 normal droughts had occurred based on 6-month SPI; 1 extreme drought, 4 severe droughts, 6 moderate droughts and 23 normal droughts had occurred based on 9-month SPI and 1 extreme drought, 3 severe droughts, 7 moderate droughts and 23 normal droughts had occurred based on 12-month SPI (Figure 5).

From the above discussion, it can be said that drought in Sylhet regions is less prominent but in the Chittagong and Hill tract regions, incidence of drought is higher and these regions were more vulnerable. Drought occurred in these regions during the years 1957, 1966, 1969, 1974, 1978, 1980, 1975, 1977, 1979, 1992, 1994, 2005, 2006 and 2008 (Figure 5).

## Conclusion

The following broad findings and conclusions are drawn from the above results and discussions.

Drought is a recurrent phenomenon in Bangladesh. Droughts occur in Bangladesh as frequently as averaging about once in 2.5 years. The north-western region of the country experienced the most severe droughts and was the most vulnerable region of the country. Droughts were also prominent in the south-western part and in the Chittagong Hill tracts also. Drought condition was found to be normal in the eastern part in maximum period. It is noted from the study that drought was normal and recurrent in Bangladesh and occurred more or less all over the country. As meteorological drought depends on only rainfall received in an area, the anomaly of rainfall was the main cause of drought.

Because of the slow-onset and end set characteristics of drought, the analysis indicates that in some cases the drought was difficult to detect and SPI values were also contradictory. In the cases of normal or slight drought, it is difficult to quantify or compare with other types of drought. In most of the cases the meteorological drought of Sylhet regions which are established as non-drought-prone areas found some results from the rainfall being irregular and variable and which may cause meteorological drought.

Bangladesh experienced major droughts during the years 1950, 1951, 1953, 1954, 1957, 1958, 1960, 1961, 1962, 1963, 1965, 1966, 1967 and 1971 before independence, and after the independence, major droughts were experienced in the years 1972, 1973, 1975, 1979, 1980, 1983, 1985, 1992, 1994, 1995, 2002, 2004, 2006, 2009 and 2011 during the period 1948–2011.

The analysis indicated that the northern regions of Rajshahi and its surroundings are more vulnerable, in south-west regions, Jessore and its surroundings areas and Bhola and the surrounding regions are vulnerable. In Sylhet division, except Srimongal the area is not vulnerable but in the south-eastern side, the districts of Rangamati, Khagrachhari, Bandarban and Teknaf are vulnerable. The Chittagong and Hill tracts regions are also vulnerable. From the central regions, Mymensingh and Faridpur are more vulnerable than other districts.
